# Mechanosensing by Gli1^+^ cells contributes to the orthodontic force‐induced bone remodelling

**DOI:** 10.1111/cpr.12810

**Published:** 2020-04-23

**Authors:** An‐Qi Liu, Li‐Shu Zhang, Ji Chen, Bing‐Dong Sui, Jin Liu, Qi‐Ming Zhai, Yan‐Jiao Li, Meng Bai, Kai Chen, Yan Jin, Cheng‐Hu Hu, Fang Jin

**Affiliations:** ^1^ State Key Laboratory of Military Stomatology & National Clinical Research Center for Oral Diseases & Shaanxi International Joint Research Center for Oral Diseases Center for Tissue Engineering School of Stomatology The Fourth Military Medical University Xi’an China; ^2^ Xi’an Institute of Tissue Engineering and Regenerative Medicine Xi’an China; ^3^ Department of Orthodontic Dentistry School of Stomatology The Fourth Military Medical University Xi’an China; ^4^ Department of Oral Implantology School of Stomatology The Fourth Military Medical University Xi’an China

**Keywords:** bone remodelling, Gli1, mechanical force, orthodontic tooth movement

## Abstract

**Objectives:**

Gli1^+^ cells have received extensive attention in tissue homeostasis and injury mobilization. The aim of this study was to investigate whether Gli1^+^ cells respond to force and contribute to bone remodelling.

**Materials and methods:**

We established orthodontic tooth movement (OTM) model to assess the bone response for mechanical force. The transgenic mice were utilized to label and inhibit Gli1^+^ cells, respectively. Additionally, mice that conditional ablate Yes‐associated protein (Yap) in Gli1^+^ cells were applied in the present study. The tooth movement and bone remodelling were analysed.

**Results:**

We first found Gli1^+^ cells expressed in periodontal ligament (PDL). They were proliferated and differentiated into osteoblastic cells under tensile force. Next, both pharmacological and genetic Gli1 inhibition models were utilized to confirm that inhibition of Gli1^+^ cells led to arrest of bone remodelling. Furthermore, immunofluorescence staining identified classical mechanotransduction factor Yap expressed in Gli1^+^ cells and decreased after suppression of Gli1^+^ cells. Additionally, conditional ablation of *Yap* gene in Gli1^+^ cells inhibited the bone remodelling as well, suggesting Gli1^+^ cells are force‐responsive cells.

**Conclusions:**

Our findings highlighted that Gli1^+^ cells in PDL directly respond to orthodontic force and further mediate bone remodelling, thus providing novel functional evidence in the mechanism of bone remodelling and first uncovering the mechanical responsive property of Gli1^+^ cells.

## 1 INTRODUCTION

Mechanical force is crucial in development and organ morphogenesis, such as the formation of the mammalian neural tube^1^ and the morphogenesis of lung branching.^2^ However, how mechanical force modulates bone remodelling remains largely unclear. Orthodontic tooth movement (OTM) depends on force‐induced periodontal ligament (PDL) and alveolar bone remodelling, including the bone deposition on tension side and resorption on compression side.^3^, ^4^ Since OTM is an in vivo model with controlled force direction, magnitude and duration, it is an ideal model for investigating how force modulates bone remodelling. The cells in periodontal ligament (PDLCs), which contain fibroblasts, osteoblasts, cementoblasts, endothelial cells and stromal/stem cells, are the primary respondents to mechanical force and key factors to alveolar bone remodelling.^5^, ^6^ Given the human periodontal ligament stromal/stem cells (hPDLSCs) are obtained and cultured easily in vitro, they have been intensively evaluated to partly elucidate the mechanism of OTM.^7^, ^8^ It has been proposed that hPDLSCs respond to mechanical force including differentiating into osteoblasts under tensile stress^5^ and promoting maturation of osteoclasts under compressive stress.^9^, ^10^ However, there are limited functional evidence about how PDLC subpopulations participate in force‐induced bone remodelling. Investigating it will extend our understanding about the cellular behaviour under mechanical force, along with providing a novel method about regulating bone remodelling.

With advances of cell tracing technique, some biomarkers such as the Lepr^+^,^11^ Scleraxis^+^ and Osterix^+^ cells^12^ have been evaluated during bone formation. Previous studies have suggested Gli1^+^ cells as progenitors contribute to the bone formation and repair process.^11^ Additionally, they could quickly amplify and migrate to participate in tissue repair process, implicating their rapid stimuli response capacity. However, whether they respond to mechanical stimuli have not been reported yet. Yes‐associated protein (Yap) is a crucial mechanical sensor that translate mechanical information into cellular biological information.^13^, ^14^ A study has demonstrated that the Gli1^+^ cells are progenitors in pulp maintaining tooth homeostasis,^15^ while whether Gli1^+^ cells exist in PDL and respond to orthodontic force remains elusive. Understanding these are beneficial to interpreting whether and how Gli1^+^ cells adapt to mechanical force and providing new insights into the behaviour of specific PDLC subpopulation influenced by orthodontic force.

The goal of this study was to examine the potential role of Gli1^+^ cells in bone remodelling induced by mechanical force. We first applied transgenic mice to investigate the proliferation and differentiation properties of Gli1^+^ cells in periodontal tissue. Then, pharmacological inhibition Gli1^+^ cells by GANT61 and genetic ablation of Gli1^+^ cells after tamoxifen treatment in *Gli1‐CreER^T2^*; *Rosa‐DTA* mice were utilized to demonstrate the indispensable role of Gli1^+^ cells during bone remodelling. Finally, by deleting *Yap* specifically in the Gli1^+^ cells, we first uncovered the Gli1^+^ cells as force‐responsive cells sense mechanical signal through Yap.

## 2 MATERIALS AND METHODS

### 2.1 Animals

The following mouse strains were obtained from the Jackson Laboratory: *Gli1‐LacZ* (JAX# 008211), *ROSA26‐eGFP‐DTA* (JAX# 006331), *Gli1‐CreER^T2^* (JAX# 007913) and *Yap^flox^* (JAX# 027929). All mice were housed in a pathogen‐free condition, maintained on the standard 12‐hour light‐dark cycle. Offspring were genotyped by PCR according to the primer sequences provided by the Jackson Laboratory, and mice were used for experiments regardless of sex at the age of 10‐12 weeks. All animal experiments were performed following the guidelines of the Intramural Animal Use and Care Committee of the Fourth Military Medical University (license number: 2018‐kq‐014).

### 2.2 Drug administration

The double transgenic mice received 100 μg/g of body weight tamoxifen in corn oil for 3 consecutive days via intraperitoneal injection. To inhibit the expression of Gli1 protein, 40 mg/kg GANT61 (Med Chem Express, USA, HY‐13901) dissolved in ethanol: corn oil (1:4) was administered in mice every other day as recommended.^16^ The vehicle was administrated to the control group.

### 2.3 Application of orthodontic devices

Mechanical force was applied in mice as previously described to move the first left maxillary molar. Briefly, orthodontic nickel‐titanium–coiled springs (0.2 mm in thickness, 1 mm in diameter, 5mm in length; Smart Technology) were ligated between the first left maxillary molar and the incisors of mice to deliver a force approximately 30 g for 7 days according to our previous study.^3^ Besides, the flowable restorative resin (3M ESPE) was used to prevent the bond failure. The mice without orthodontic devices served as control. All mice received soft diet after operation.

### 2.4 Micro‐computed tomography (Micro‐CT) analysis

Freshly dissected maxillae were collected and scanned by Micro‐CT (Siemens Inveon, Germany). The sagittal and horizontal images were acquired through three‐dimensional reconstructions. OTM distance was measured as previously described.^17^

### 2.5 Immunofluorescence staining

For immunofluorescence staining, the decalcified samples were embedded and frozen in optimum cutting temperature compound (OCT), and sliced into 20 μm thick sections (CM1950; Leica, Germany).

For immunostaining, sections were permeabilized in 1% Triton X‐100 (Sigma‐Aldrich, USA) for 5 minutes, blocked in goat serum (Sigma‐Aldrich, USA) at room temperature for 30 minutes, and incubated with the primary antibodies overnight at 4℃. The primary antibodies were as follows: beta‐galactosidase (β‐gal; Abcam, ab9361, UK; 1:200), CD31 (R&D Systems, FAB3628G, USA; 1:100), Rankl (Abcam, ab40539, UK; 1:100), Runt‐related transcription factor 2 (Runx2, Cell Signaling Technology, #12556, USA; 1:200), tartrate‐resistant acid phosphatase (Trap; Abcam, ab191406, UK; 1:100), active‐Yap (Abcam, ab205270, UK; 1:100) and Yap (Cell Signaling, #14074, USA; 1:100). Then, sections were incubated with appropriate secondary antibodies (Jackson, USA; 1:200) for 1.5 hours at room temperature.

### 2.6 Haematoxylin and eosin (HE) staining and tartrate‐resistant acid phosphatase (Trap) staining

Freshly dissected maxillae were collected and fixed in 4% paraformaldehyde (PFA; Sigma‐Aldrich, USA) solution for 6h at 4°C. Samples were decalcified with 0.5M ethylenediaminetetraacetic acid (EDTA; MP Biomedicals, USA) at 4℃. Decalcified samples were then embedded with paraffin and sliced in the horizontal or sagittal plane for haematoxylin and eosin (H&E) (Leica, Germany) and tartrate‐resistant acid phosphatase (Trap) staining. Sections were stained for Trap using a commercial kit (Wako, Japan, Code No. 294‐67001) according to the manufacturer's protocol. Trap^+^ multinucleated cells containing at least three nuclei were identified as osteoclasts. Trap^+^ osteoclasts attached to alveolar bone surfaces were counted in the mesial sides of OTM.

### 2.7 Image acquisition and quantitative analysis

Immunofluorescence staining sections were analysed using a Nikon laser scanning confocal microscope (A1 Plus, Nikon, Japan). Qualifications of images were carried out with ImageJ (Media Cybernetics, USA).

### 2.8 Statistical analysis

Statistical analysis was performed with GraphPad Prism 5.0. Comparison between groups was statistically analysed by two‐tailed unpaired Student's *t* test. Values of *P* less than 0.05 were considered statistically significant. All data were expressed as mean (±SD).

## 3 RESULTS

### 3.1 Gli1^+^ cells in PDL participate in the force‐triggered bone remodelling

**Figure 1:**
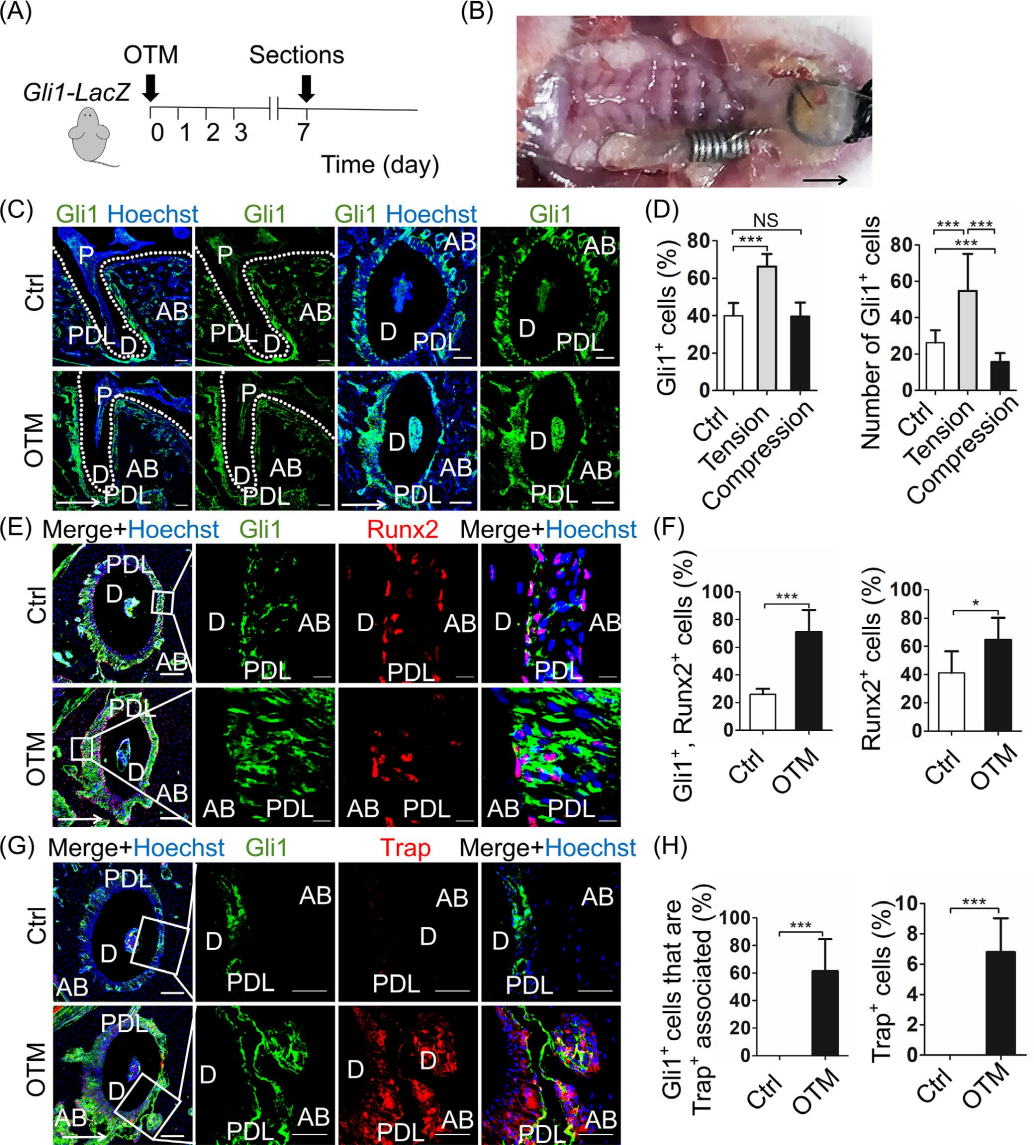
Gli1^+^ cells in PDL participate in the force‐triggered bone remodelling. A, Experimental design: *Gli1‐LacZ* mice sacrificed after 7 days of OTM (OTM). The mice without surgery were used as control (Ctrl). B, Intraoral representation of the application of orthodontic devices in mice. Coiled springs are ligated between the first left maxillary molar and the incisors, then fixed by resin. C, The sagittal and horizontal sections of distobuccal root of first maxillary molar display the cytoplasmic distribution of Gli1^+^ cells (green). Scale bar: 100 µm. D, Compared with the Ctrl group, the number of Gli1^+^ cells and the percentage of Gli1^+^ cells in periodontal ligament in OTM group expand on tension side. On compression side, the number of Gli1^+^ cells decrease in the OTM group, but the percentage of Gli1^+^ cells in periodontal ligament has no statistic difference between the two groups. ***P < .005; NS, *P* > .05; n = 6. E, Low magnification (left panel) shows Gli1 (green) co‐expression with Runx2 (red). Scale bar: 100 µm. Boxed areas show high magnification that Gli1^+^ cells express Runx2 in nuclear and increase on tension side. Scale bar: 10 µm. F, The proportion of both Gli1^+^, Runx2^+^ cells and Runx2^+^ cells elevate on tension side, indicating the Gli1^+^ cells directly participate in osteogenesis. ***P < .005; *P < .05; n = 6. G, Low magnification (left panel) shows the co‐staining of Gli1 (green) and Trap (red). Scale bar: 100 µm. Boxed areas show high magnification that Gli1^+^ cells are adjacent to Trap^+^ osteoclasts but barely have co‐localization on compression side, indicating the close relationship between Gli1^+^ cells and osteoblasts. Scale bar: 50 µm. H, The proportion of Gli1^+^ that adjacent to Trap^+^ cells and Trap^+^ cells both significantly increase on compression side. ***P < .005; n = 5. Arrows indicate the direction of tooth movement; P: pulp; D: dentine; PDL: periodontal ligament; AB: alveolar bone

**Figure A1:**
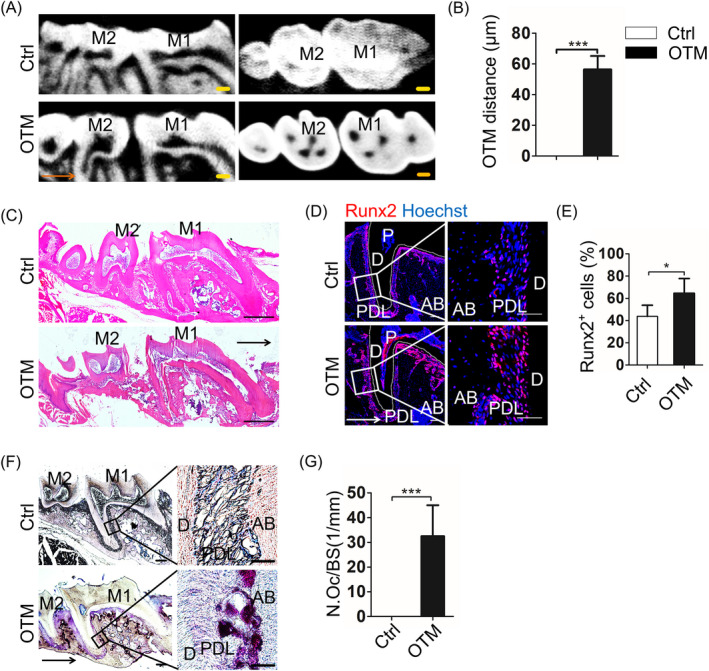
Evaluation of OTM model. A, Micro‐computed tomography analysis displays the sagittal and horizontal images of tooth. Scale bar: 200 µm. M1: the first maxillary molar; M2: the second maxillary molar. B, The data show the distance of tooth movement increase in the OTM group (OTM). The tooth movement in mouse tooth without orthodontic force (Ctrl) is undetectable. ***P < .005; n = 5. C, Haematoxylin‐eosin staining exhibits the sagittal section of tooth in Ctrl and OTM group. The alveolar bone in the OTM group shows more spaces of bone marrow. Scale bar: 250 µm. D, Immunofluorescence staining demonstrates the nuclear localization of Runx2 (red). Scale bar: 100µm. Boxed areas are shown magnified to the right. Scale bar: 50 µm. E, The percentage of Runx2^+^ cells increases on tension side. *P < .05; n = 6. F, Tartrate‐resistant acid phosphatase (TRAP) staining displays the sagittal section of tooth in the Ctrl and OTM group. Scale bar: 50µm; arrowheads indicate osteoclasts on alveolar bone surfaces. Boxed areas are shown magnified to the right. Scale bar: 10 µm. G, The corresponding parameter of number of osteoclasts per bone surface (N.Oc/BS) demonstrates that the number of TRAP^+^ cells significantly increases in the OTM group. ***P < .005; n = 5. Arrows indicate the direction of tooth movement; P: pulp; D: dentine; PDL: periodontal ligament; AB: alveolar bone

**Figure A2:**
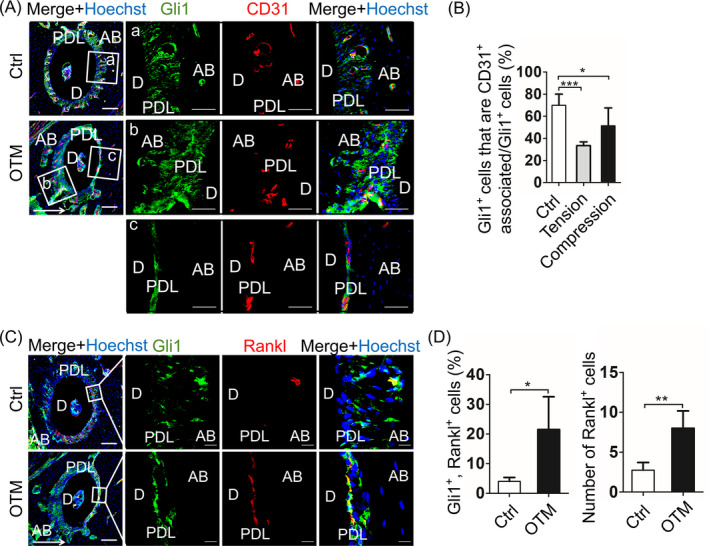
Gli1^+^ cells are associated with blood vessel and express Rankl under compressive force. A, In *Gli1‐LacZ* mice, immunofluorescence staining demonstrates Gli1^+^ cells (green) are associated with CD31^+^ endothelial cells (red) in tooth with (OTM) or without (Ctrl) orthodontic force. Low magnification of roots is exhibited to the left. Scale bar: 100 µm. Boxed areas show high magnification that most Gli1^+^ cells around CD31^+^ endothelial cells in Ctrl group (a). The Gli1^+^ cells distribute not only mainly around CD31^+^ cells, but also the whole periodontal ligament (b, c). (B) The percentage of Gli1^+^ cells that associated with CD31^+^ endothelial cells decreases on tension and compression side, but the percentage of Gli1^+^ cells increases on tension side, indicating Gli1^+^ cells may proliferate and migrate from vessels in OTM group. b, c,. Scale bar: 50 µm. ***P < .005; *P < .05; n = 5. C, The immunofluorescence staining shows co‐localization of Gli1 and the classical osteoclast differentiation factor Rankl. Low magnification of roots is exhibited to the left. Scale bar: 100 µm. Boxed areas show high magnification that co‐localization of Gli1 and Rankl. D, Compared to Ctrl, the proportion of Gli1^+^, Rankl^+^ cells and the number of Rankl^+^ cells both increase on compression side. Scale bar: 10 µm. **P < .001; *P < .05; n = 4. Arrow indicates the direction of tooth movement; D: dentine; PDL: periodontal ligament; AB: alveolar bone

To investigate the expression pattern of Gli1^+^ cells during tooth movement, we first used *Gli1‐LacZ* mice, which labelled Gli1 in cytoplasm to establish the mouse OTM model (OTM) (Figure 1A,B). The mice without orthodontic force loaded were used as control (Ctrl). The mesial movement of maxillary first molar was detected from micro‐CT analysis (Appendix Figure A1A,B; n = 5; *P* < .005). HE staining showed PDL with narrow space on compression side and wide space on tension side (Appendix Figure A1C). Since the OTM depends on bone formation and resorption on the tension and compression side respectively, we evaluated the bone remodelling through early osteogenic differentiation marker Runt‐related transcription factor 2 (Runx2) and osteoclast marker tartrate‐resistant acid phosphatase (Trap). After 7 days of OTM, we observed an increase in Runx2 nuclear localization on the tension side (Appendix Figure A1D,E; n = 6; *P* < .05), as well as the Trap^+^ cells on the compression side (Appendix Figure A1F, G; n = 5; *P* < .005), consisting with previous studies.^3^ Then, we investigated the expression pattern of Gli1^+^ cells during OTM. In the Ctrl group, Gli1^+^ cells were not only associated with CD31^+^ vasculature, but also distributed on the surface of both alveolar bone and cementum (Appendix Figure A2A,B; n = 6). During tooth movement, the number of Gli1^+^ cells and the percentage of Gli1^+^ cells in periodontal ligament in the OTM group expanded on tension side (Figure 1C,D; n = 6; *P* < .005) and migrated from vasculature (Appendix Figure A2A,B; n = 5; *P* < .005). On compression side, though the number of Gli1^+^ cells decrease in the OTM group when compared to the Ctrl group, the proportion of Gli1^+^ cells in periodontal ligament has no statistic difference between the two groups (Figure 1C,D; n = 6; Appendix Figure A2A,B; n = 5; *P* > .05), indicating Gli1^+^ cells may participate in maintaining the homeostasis of PDL. In addition, we evaluated the expression of Runx2 and Trap with Gli1. High‐magnification images of co‐staining indicated most Gli1^+^ cells expressed Runx2 in nucleus, especially as thin layers of cells lining on the surface of alveolar bone and cementum (Figure 1E,F; n = 6; *P* < .005). Although we barely detected co‐localization of Gli1 and Trap, we found Gli1^+^ cells were always adjacent to Trap^+^ osteoclasts, implying the close relationship between Gli1^+^ cells and osteoclasts (Figure 1G,H; n = 5; *P* < .005). The percentage of Runx2^+^ or Trap^+^ cells significantly increased on the tension or compression side, respectively (Figure 1F; n = 6; *P* < .05; Figure 1H; n = 5; *P* < .005). Furthermore, we analysed the classical osteoclast differentiation factor receptor activator of nuclear factor‐κ B ligand (Rankl), which is secreted by osteoblasts and osteocytes.^18^ The immunofluorescence staining showed co‐localization of Gli1 and Rankl. Besides, compared to Ctrl, the proportion of Gli1^+^, Rankl^+^ cells and the number of Rankl^+^ cells both increased on compression side (Appendix Figure A2C,D; n = 4; *P* < .05, *P* < .01). To our knowledge, our findings first proposed that Gli1^+^ cells in PDL can respond to orthodontic force‐induced bone remodelling.

### 3.2 Pharmacologic inhibition of Gli1^+^ cells gives rise to suppression of bone remodelling

**Figure 2:**
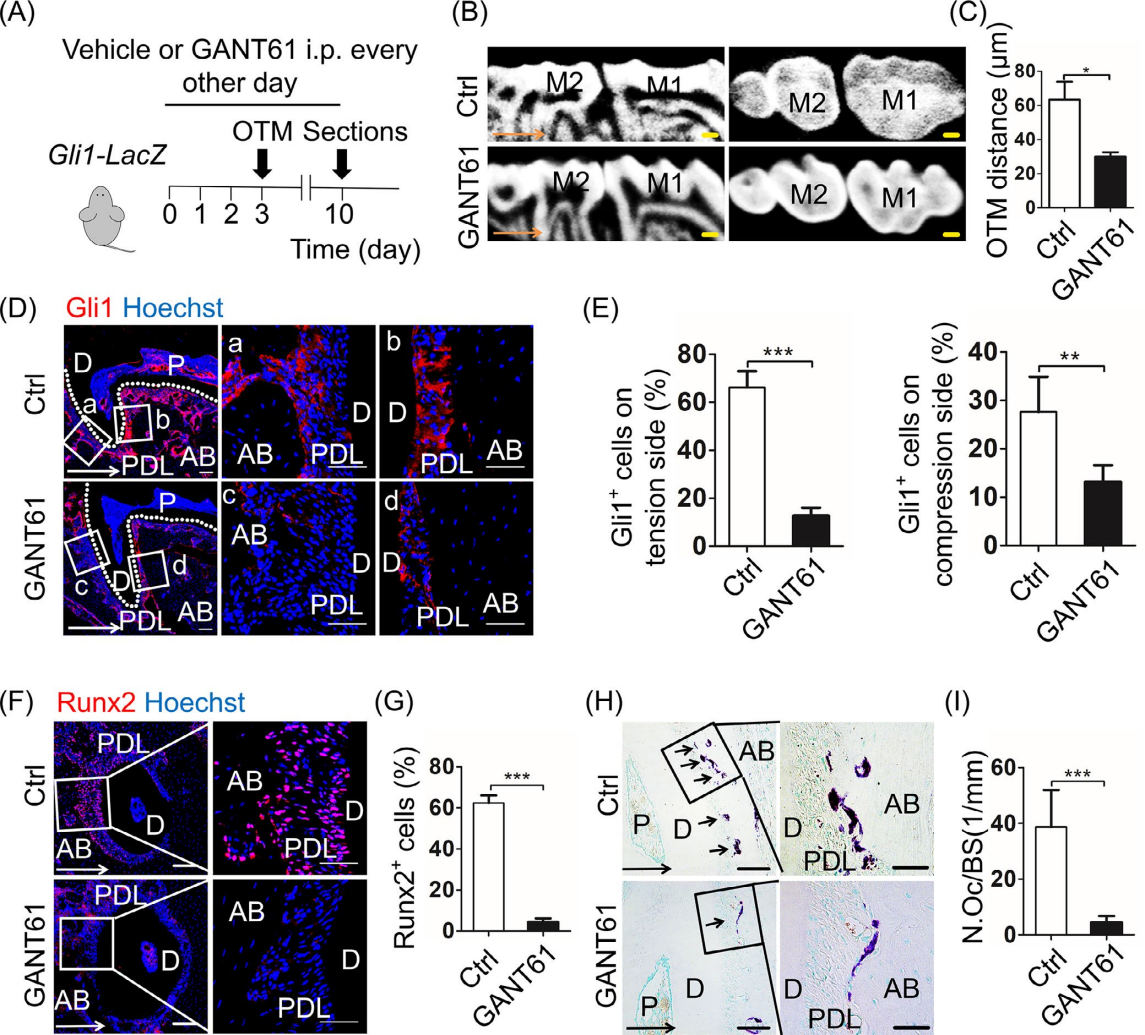
Pharmacologic inhibition of Gli1^+^ cells gives rise to suppression of bone remodelling. A, Experimental design: 40 mg/kg GANT61 or vehicle was administrated to *Gli1‐LacZ* mice every other day three days before OTM. After 7 days of OTM, the maxillary was harvested. B, The sagittal and horizontal images in the micro‐computed tomography demonstrate mice injected with GANT61 (GANT61) or vehicle (Ctrl). Scale bar: 200 µm. M1: the first maxillary molar; M2: the second maxillary molar. C, The measurement of OTM distance shows GANT61 injection inhibits tooth movement. *P < .05; n = 5. D, Low magnification (left panel) shows the expression of Gli1 (red) between Ctrl and GANT61 group. Scale bar: 100 µm. Boxed areas respectively show high magnification of Ctrl group (a, b) and GANT61 group (c, d). Scale bar: 50 µm. E, In comparison with the Ctrl group, the percentage of Gli1^+^ cells in the GANT61 group reduces on two sides. ***P < .005; **P < .01; n = 6. F, Immunofluorescence staining displays expression of Runx2 (red). Scale bar: 100µm. Boxed areas are shown magnified to the right. Scale bar: 50 µm. G, Runx2^+^ cells significantly decrease after GANT61 treatment on tension side. ***P < .005; n = 6. H, Tartrate‐resistant acid phosphatase (TRAP) staining on compression side. Scale bar: 50µm; arrowheads indicate osteoclasts on alveolar bone surfaces. Boxed areas are shown magnified to the right. Scale bar: 10 µm. I, The corresponding parameter of number of osteoclasts per bone surface (N.Oc/BS) demonstrates TRAP^+^ cells significantly decrease after GANT61 treatment. ***, *P* < .005; n = 6. Arrows indicate the direction of tooth movement; P: pulp; D: dentine; PDL: periodontal ligament; AB: alveolar bone

**Figure A3:**
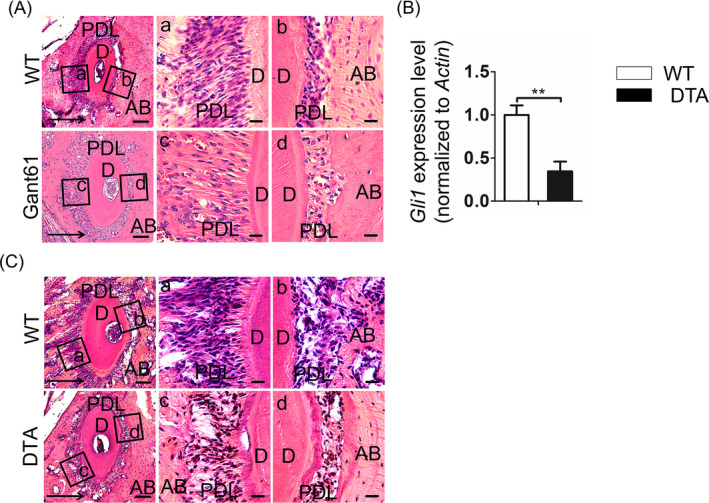
Inhibition of Gli1^+^ cells reduces the number cells on tension side, along with total *Gli1* gene expression. A, Haematoxylin‐eosin staining shows the roots from mice injected with GANT61 (GANT61) or vehicle (Ctrl). Low magnification (left panel) exhibits the horizontal sections of roots from Ctrl and GANT61 group. Scale bar: 100 µm. Boxed areas respectively show high magnification of Ctrl and GANT61 group. Comparing with Ctrl group, GANT61 group displays fewer cells but more matrix on tension side (a, c). No significant histological difference has been detected on compression side (b, d). Scale bar: 20 µm. B, Compared with wild‐type group (WT), the *Gli1* gene expression of periodontal tissue reduces in DTA group. **P < .01; n = 3. C, Haematoxylin‐eosin staining demonstrates the roots from *Gli1‐CreER^T2^*; *Rosa*‐*DTA* mice (DTA) and wild‐type mice (WT). Low magnification (left panel) shows the horizontal sections of roots from WT and DTA group. Scale bar: 100 µm. Boxed areas respectively display high magnification of WT and DTA group. Comparing with WT group, DTA group displays fewer cells on tension side (a, c). On compression side, DTA group shows less bone resorption pits (b, d). Scale bar: 20 µm. Arrows indicate the direction of tooth movement; D: dentine; PDL: periodontal ligament; AB: alveolar bone

To investigate the function of Gli1^+^ cells in regulating force‐induced bone remodelling, we administrated GANT61, a small molecule efficiently blocked Gli1 protein, to *Gli1‐LacZ* mice.^19^, ^20^ Treatment with GANT61 (GANT61) or vehicle (Ctrl) was 3 days before surgery to inhibit Gli1 expression previously (Figure 2A). Micro‐CT analysis revealed only a minor tooth movement in the GANT61 group (Figure 2B,C; n = 5; *P* < .05). In comparison with Ctrl, GANT61 group exhibited similar PDL reaction through HE staining, such as narrowing on the compression side and widening on the tension side. (Appendix Figure A3A). Application of GANT61 resulted in decrease in both Gli1^+^ cells (Figure 2D,E; n = 6; *P* < .005) and Runx2^+^ cells (Figure 2F, G; n = 6; *P* < .005), which are consistent with previously reports. On the compression side, the Trap^+^ cells also reduced in the GANT61 group (Figure 2H,I; n = 6; *P* < .005). The findings above demonstrated that suppression of Gli1 protein inhibited tooth movement and bone remodelling, implying that Gli1^+^ cells are critical in regulating alveolar bone remodelling.

### 3.3 Genetic ablation of Gli1^+^ cells leads to arrest of bone remodelling

**Figure 3:**
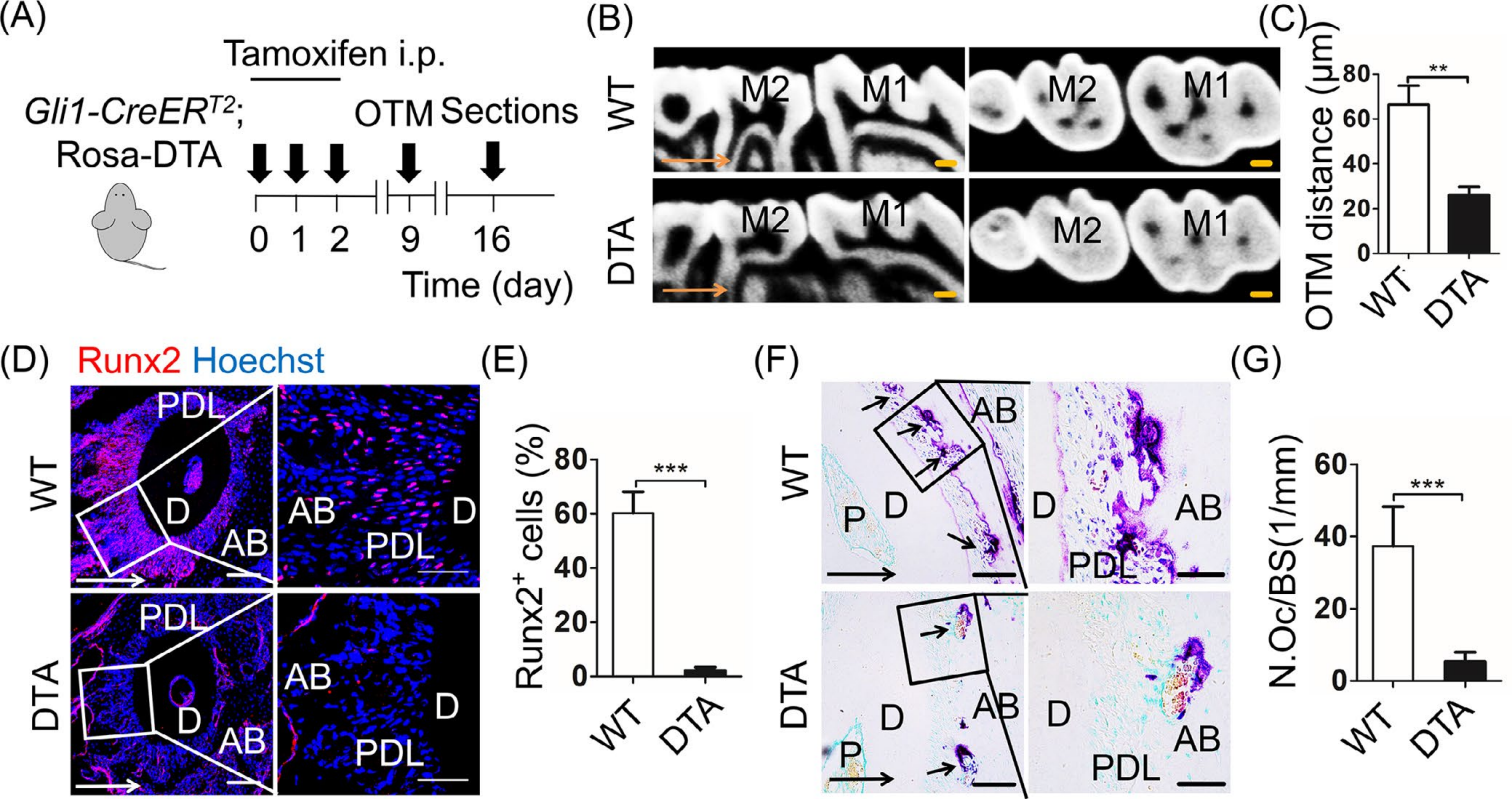
Genetic ablation of Gli1^+^ cells leads to arrest of bone remodelling. A, Experimental design: *Gli1‐CreER^T2^*; *Rosa*‐*DTA* mice (DTA) or wild‐type mice (WT) were injected with tamoxifen (100 μg/g body weight intraperitoneally [i.p.]) for 3 consecutive days. Orthodontic force was applied to mice at the 9th day after the first tamoxifen dose. After 7 days of OTM, the maxillary was harvested. B, The sagittal and horizontal images in the micro‐computed tomography demonstrate the shorter distance of DTA group, compared with WT. Scale bar: 200 µm. M1: the first maxillary molar; M2: the second maxillary molar. C, The measurement of OTM distance shows ablation of Gli1^+^ cell inhibits tooth movement. **P < .01; n = 5. D, Immunofluorescence staining displays expression of Runx2 (red). Scale bar: 100µm. Boxed areas are magnified to the right. Scale bar: 50 µm. E, Runx2^+^ cells significantly decrease in the DTA group on tension side. ***P < .005; n = 6. F, Tartrate‐resistant acid phosphatase (TRAP) staining on compression side. Scale bar: 50µm; arrowheads indicate osteoclasts on alveolar bone surfaces. Boxed areas are shown magnified to the right. Scale bar: 10 µm. G, The corresponding parameter of number of osteoclasts per bone surface (N.Oc/BS) exhibits TRAP^+^ cells significantly decrease in DTA mice. ***P < .005; n = 6. Arrows indicate the direction of tooth movement; P: pulp; D: dentine; PDL: periodontal ligament; AB: alveolar bone

It has been suggested that GANT61 not only effectively inhibits Gli1 protein, but also represses downstream factors of Hh signaling.^19^ To further confirm the impact of Gli1^+^ cells on OTM triggered bone remodelling, we crossed *Gli1‐CreERT2* mice with *ROSA26‐eGFP‐DTA* mice to generate *Gli1‐CreER^T2^; Rosa‐DTA* mice (DTA), which genetically ablated Gli1^+^ cells. Specifically, we applied tamoxifen to either DTA mice or wild‐type mice (WT) 3 consecutive days and waited 7 days for the expression of DTA sufficiently, then applied orthodontic force for 7 days (Figure 3A). To confirm the effectiveness of the cell ablation technique, we used qRT‐PCR to analyse the *Gli1* gene expression level of periodontal tissue between DTA and WT mice. The results showed a striking reduction of the expression level of *Gli1* gene in DTA mice (Appendix Figure A3B; n = 3; *P* < .001). Even the DTA model was not entirely efficient, this incomplete Gli1^+^ cell suppression resulted in the arrest of OTM, which is consistent with GANT61 administration (Figure 3B,C; n = 5; *P* < .001). HE staining demonstrated the PDL of DTA mice displayed similar phenotype to WT (Appendix Figure A3C). Then, we evaluated the expression of Runx2 and Trap as before. To our surprise, the Runx2^+^ cells were obviously eliminated in DTA mice (Figure 3D,E; n = 6; *P* < .005). The number of Trap^+^ osteoclasts was decreased in DTA mice on the compression side (Figure 3F, G; n = 6; *P* < .005). These data confirmed that Gli1^+^ cells in PDL contribute to force‐mediated OTM and alveolar bone remodelling.

### 3.4 Gli1^+^ cells are mechanical sensors through Yap activation

**Figure 4:**
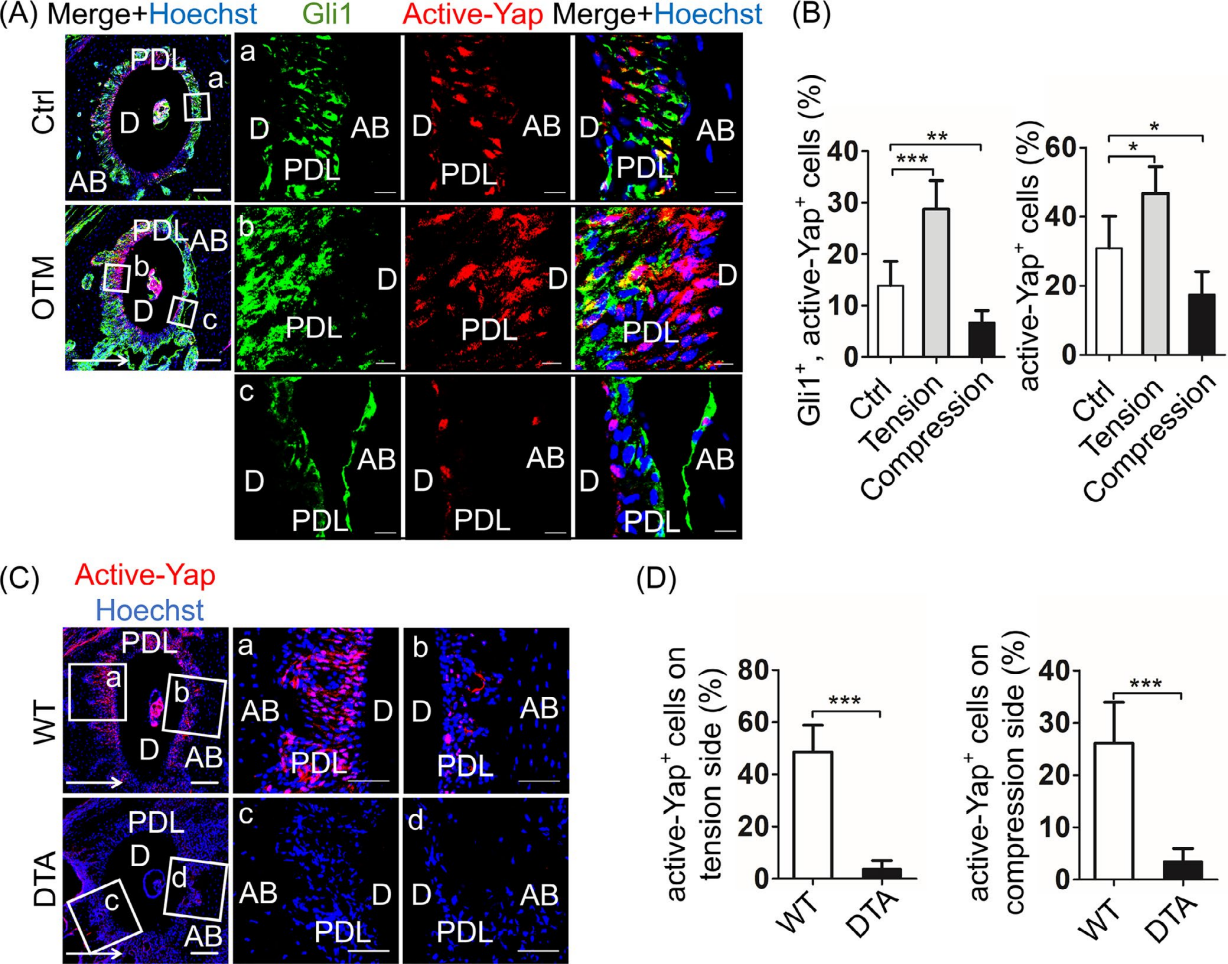
Gli1^+^ cells are mechanical sensors through Yap activation. A, In *Gli1‐LacZ* mice, immunofluorescence staining shows Gli1 (green) co‐expression with active‐Yap (red) in tooth with (OTM) or without (Ctrl) orthodontic force. Low magnification of roots exhibit to the left. Scale bar: 100 µm. Boxed areas show high magnification that partly cytoplasmic expression Gli1^+^ cells express active‐Yap in nuclear in the Ctrl group (a). The co‐localization increases on tension side (b) and decreases on compression side (c). Scale bar: 10 µm. B, Compared with the Ctrl group, the higher proportion of both Gli1^+^, active‐Yap^+^ cells and active‐Yap^+^ cells on tension side, while lower on compression side. ***P < .005; **P < .01; *P < .05; n = 6. C, Inhibiting Gli1^+^ cells in *Gli1‐CreER^T2^*; *Rosa*‐*DTA* mice, active‐Yap^+^ cells (red) distinctly reduce on tension (a, c) and compression side (b, d), compared with wild‐type mice (WT). D, Comparing to the WT group, the percentage of active‐Yap^+^ cells in the DTA group distinctly drops on tension and compression side. ***P < .005; n = 6. Arrows indicate the direction of tooth movement; P: pulp; D: dentine; PDL: periodontal ligament; AB: alveolar bone

The data so far have indicated that Gli1^+^ cells are indispensable PDLC population during OTM, but the underlying mechanism remains unknown. Given PDLCs can sense force through Yap expression, along with osteogenesis in vitro,^21^ we hypothesized that Gli1^+^ cells respond to force and regulate OTM through Yap activation. Yap is a classical transcription factor that responds to mechanical force.^2^ After being activated, dephosphorylated Yap transfers into nucleus and activates the transcription of downstream genes, regulating cell behaviours.^22^ In this study, we first detected the expression pattern of Gli1 and active‐Yap using *Gli1‐LacZ* mice. Immunofluorescence staining exhibited that a part of Gli1^+^ cells strongly expressed active‐Yap, which mainly accumulated in nucleus, in the control group (Ctrl) (Figure 4Aa). After 7 days of OTM (OTM), the proportion of both Gli1^+^, active‐Yap^+^ cells and active‐Yap^+^ cells increased on tension side and decreased on compression side (Figure 4Ab,C,B; n = 6; *P* < .05), implying the expression of active‐Yap is similar to Gli1 during OTM. Next, we analysed the relationship between active‐Yap and Gli1. Compared with wild‐type OTM mice (WT), ablation of Gli1 (DTA) significantly disrupted the expression of active‐Yap (Figure 4C,D; n = 6; *P* < .005). The data above showed the PDL responds to force by activating Yap, which relies on the existence of Gli1^+^ cells, implicating the indispensable role of Gli1^+^ cells in responding to orthodontic force.

### 3.5 Genetic ablation of Yap specific in Gli1^+^ cells inhibits bone remodelling

**Figure 5:**
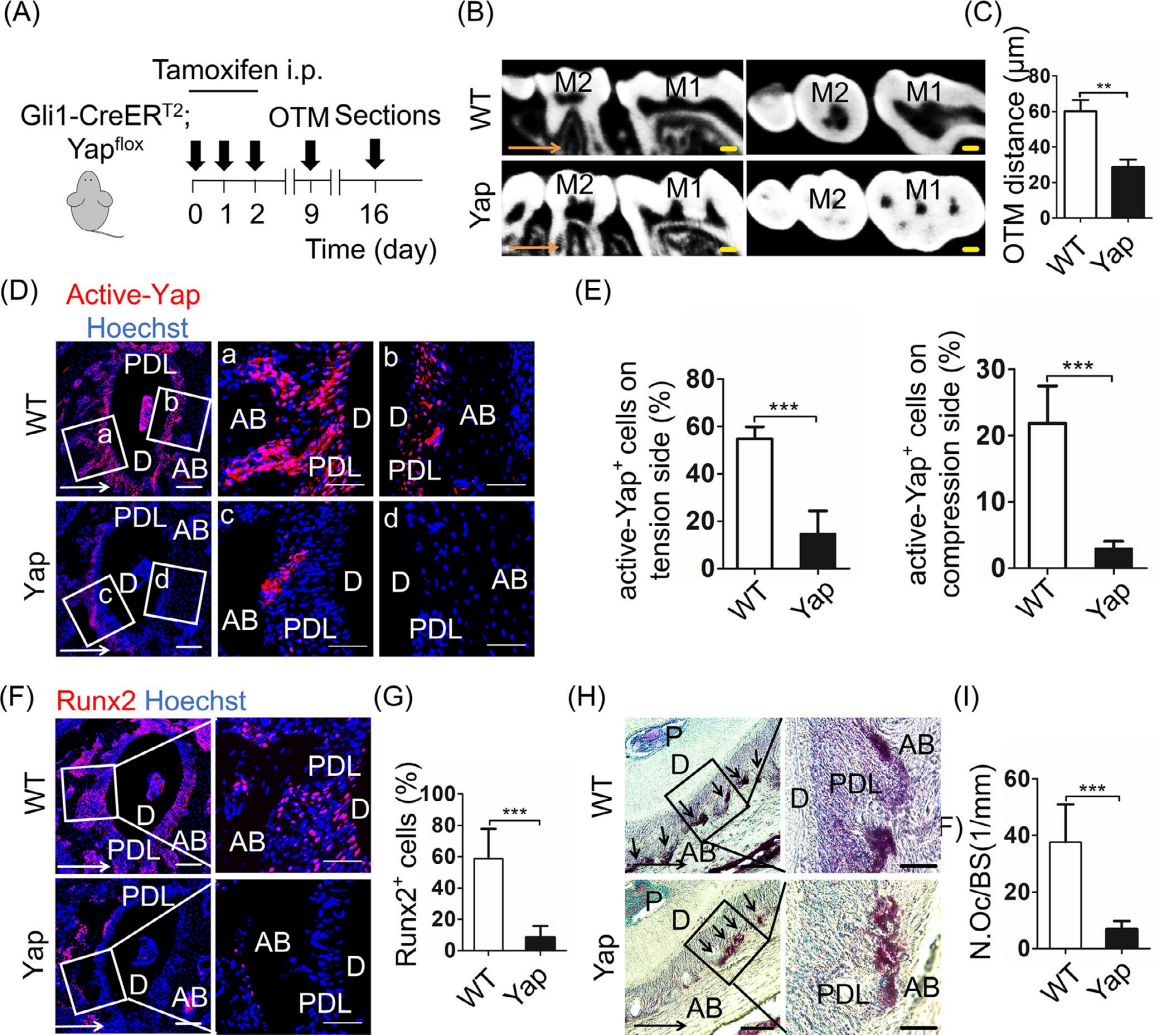
Genetic ablation of *Yap* specific in Gli1^+^ cells inhibits bone remodelling. A, Experimental design: *Gli1‐CreER^T2^*; *Yap* mice (Yap) or wild ‐type mice (WT) were injected with tamoxifen (100 μg/g body weight intraperitoneally [i.p.]) for 3 consecutive days. Orthodontic force was applied to mice at the 9th day after the first tamoxifen dose. After 7 days of OTM, the maxillary was harvested. B, The sagittal and horizontal images in the micro‐computed tomography show the distance of OTM in the Yap group is shorter than the WT group. Scale bar: 200 µm. M1: the first maxillary molar; M2: the second maxillary molar. C, The data demonstrate specific ablation of the *Yap* gene in Gli1^+^ cells suppresses tooth movement. **P < .01; n = 5. D, Compared with the WT group, inhibiting *Yap* gene in the Yap group distinctly reduces the expression of active‐Yap^+^ cells (red) on tension (a, c) and compression side (b, d). E, The percentage of active‐Yap^+^ cells in Yap group significantly drops on tension and compression side. ***P < .005; n = 5. F, Immunofluorescence staining shows the expression of Runx2 (red). Scale bar: 100µm. Boxed areas are shown magnified to the right. Scale bar: 50 µm. G, On tension side, the proportion of Runx2^+^ cells significantly decreases in Yap group when compared to WT group. ***P < .005; n = 6. H, Tartrate‐resistant acid phosphatase (TRAP) staining on compression side. Scale bar: 50µm; arrowheads indicate osteoclasts on alveolar bone surfaces. Boxed areas are shown magnified to the right. Scale bar: 10 µm. I, The corresponding parameter of number of osteoclasts per bone surface (N.Oc/BS) demonstrates TRAP^+^ cells significantly reduce in Yap group. ***P < .005; n = 6. Arrows indicate the direction of tooth movement; P: pulp; D: dentine; PDL: periodontal ligament; AB: alveolar bone

**Figure A4:**
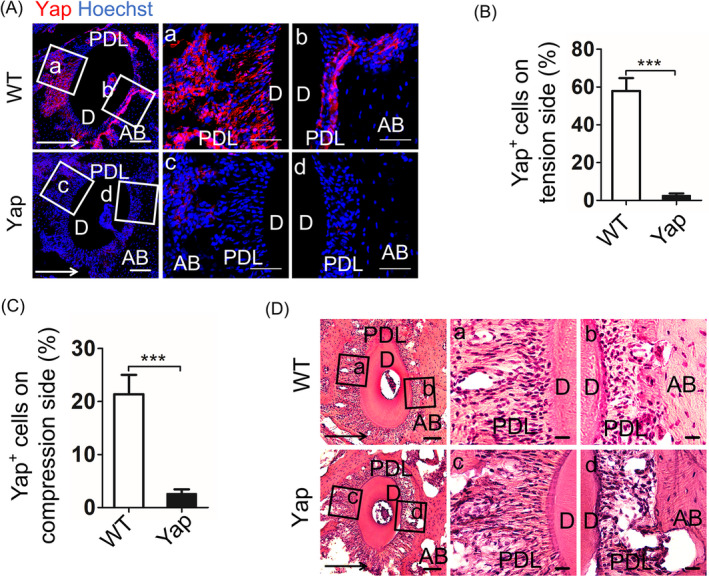
Inhibition of the *Yap* gene in Gli1^+^ cells suppresses the expression of Yap protein. A, Immunofluorescence staining exhibits the expression of total Yap protein in *Gli1‐CreER^T2^*; *Yap^flox^* mice (Yap) and wild‐type mice (WT). Low magnification of roots exhibits to the left. Scale bar: 100 µm. Boxed areas respectively show high magnification of WT and Yap group. The total Yap protein expresses in the whole periodontal ligament during tooth movement in WT group (a, b), but significantly reduces in Yap group (c, d). Scale bar: 20 µm. B, The percentage of Yap^+^ cells in Yap group distinctly drops on tension side. ***P < .005; n = 5. C, The percentage of Yap^+^ cells in Yap group significantly decreases on compression side. ***P < .005; n = 5. D, Haematoxylin‐eosin staining shows the roots of WT and Yap group. Boxed areas on tension side (a, c) and compression side (b, d) exhibit no significant histological difference. Arrows indicate the direction of tooth movement; D: dentine; PDL: periodontal ligament; AB: alveolar bone.

To test whether Yap in Gli1^+^ cells is involved in the process of OTM, we applied OTM in *Gli1‐CreER^T2^; Yap* transgenic mice with specific knock of the *Yap* gene in Gli1^+^ cells after tamoxifen application (Yap). The wild‐type mice with OTM were used as control (WT) (Figure 5A). The micro‐CT showed a suppression of tooth movement in Yap mice when compared to the WT group (Figure 5B,C; n = 5; *P* < .001). From the immunofluorescence staining, we detected a significant reduction of not only active‐Yap^+^ cells (Figure 5D,E; n = 5; *P* < .005) but also total Yap^+^ cells in the Yap group after tamoxifen induction (Appendix Figure A4A–C; n = 5; *P* < .005). Subsequently, we found the proportion of Runx2^+^ cells on tension side (Figure 5F, G; n = 6; *P* < .005) and the number of Trap^+^ cells on compression side (Figure 5H,I; n = 6; *P* < .005) decreased in Yap mice, consisting with the data from GANT61 and DTA mice. However, we did not detect a distinct histological difference between Yap and WT group (Appendix Figure A4D). The findings showed for the first time that specific knock of the *Yap* gene in Gli1^+^ cells arrested alveolar bone remodelling. Thus, we suppose that Yap act as a mechanical sensor in Gli1^+^ cells responding to mechanical force and regulating behaviour of Gli1^+^ cells.

## 4 DISCUSSION

Under orthodontic force, cells in PDL participate in bone formation on tension side and resorption on compression side, making it possible for tooth movement.^9^, ^23^, ^24^ Thus, the detectable tooth movement enacts indicator for bone remodelling. The present study proposes for the first time that Gli1^+^ cells as force‐responsive PDLC subpopulation are required for bone remodelling. Specifically, once Gli1^+^ cells in PDL sense force, they activate Yap to modulate downstream gene expression, contributing to bone remodelling.

Mechanical force regulates bone remodelling, which orchestrates bone formation and bone resorption during physiologic and pathologic conditions. For instance, a higher level of physical activity is beneficial to preventing bone loss for perimenopausal women.^25^ Spaceflight leads astronauts to significant bone loss, which demonstrates that weightlessness results in decrease in bone mass.^26^ Given that orthodontic force induces alveolar bone remodelling leading to tooth movement, OTM is an ideal animal model exploring how osseous tissue responds to controlled force. In general, application of a force on tooth initially narrows the PDL on compression side, then promoting differentiation of osteoclasts, the PDL regaining the width after osteoclastic removal of bone.^9^ On tension side, the width of PDL increases with cell proliferation, then the new bone deposition on alveolar surface, leading the width of PDL to normal limits.^23^, ^27^ Given that hPDLSCs are obtained and cultured easily in vitro, they are widely applied in investigating the mechanism of OTM.^6^ For example, hPDLSCs have the property of osteogenesis.^24^ Besides, they can express receptor activator of nuclear factor κB ligand (RANKL) to combine with the receptor activator of nuclear factor κB (RANK), which is expressed by osteoclast progenitors, inducing osteoclastogenesis.^18^ However, there still lack functional evidence of PDLC subpopulation in OTM. Gli1^+^ cells have been suggested as mesenchymal progenitors giving rise to odontoblasts in pulp and osteoblasts in bone.^11^, ^15^ In this study, we first found that Gli1^+^ cells in the periodontal tissue not only reside around blood vessels (Appendix Figure A2Aa), but also reside in the front of bone and cementum formation participating in the physiological periodontal remodelling that may correlate with masticatory force (Figure 1C). Furthermore, we showed pharmacological inhibition and genetic ablation of Gli1^+^ cells in two mice OTM models lead to the arrest of OTM. A study has proposed that sensory nerve section results in suppression of OTM.^28^ Given that sensory nerve activates Gli1 expression by Shh protein secretion,^15^ our discovery partly explains the phenomena. To our knowledge, our study is the first to find that Gli1^+^ cells are directly responsible for OTM. The findings provide a new insight into the mechanism of how PDLC subpopulation mediates the process of bone remodelling in vivo. Furthermore, the application of Gli1 protein inhibitor GANT61 may be a new pharmacological target for the anchorage tooth control.

Force has been demonstrated to influence cellular processes including cell shape changes, proliferation, migration and differentiation.^22^, ^29^, ^30^ Extensive studies have presented that cells sense the mechanical force through cell membrane or cytoskeleton then converts the mechanical signals into biochemical signals to induce downstream gene transcription, thereby leading to cell adaptive behaviour.^22^, ^31^, ^32^ For instance, after sensing tensile force, PDLSCs increase Runx2 expression, exhibiting higher osteogenic differentiation ability.^24^ With the advances of cell tracing technique, an increasing number of stromal/stem cells markers, such as NG2^+^ cells,^33^ Sox2^+^ cells^34^ and Gli1^+^ cells,^15^ in tooth are used to elucidate the cell behaviour during tooth development or injury. For example, Sox2^+^ cells produce ameloblasts and other epithelial cell lineages of the incisor.^34^ The NG2^+^ cells, which mark pericytes, can differentiate into odontoblast‐like cells in mouse incisor.^15^, ^33^ However, how force influences the fate and behaviour of specific subpopulations of stromal/stem cells in vivo largely remains unknown. In this study, we detected Gli1^+^ cells expanded and expressed Runx2 on tension side, indicating the Gli1^+^ cells proliferate and differentiate into osteoblastic cells under tensile force (Figure 1E). Further, we investigated osteoclast marker Trap and osteoclastogenesis‐inducing factor Rankl, which is mainly secreted by osteoblasts and osteocytes. Osteoblast and osteoclast coordination determines balance of bone metabolism. In terms of mechanism, Opg/Rank/Rankl system is a classical theory to interpret the bone metabolism. Among them, osteoprotegerin (Opg) and Rankl are expressed by osteogenic lineages to competitive binding with Rank, which is expressed by osteoclast progenitor. Under compressive force, osteoblasts secrete Rankl to combine with Rank, inducing osteoclastogenesis.^18^, ^27^ In the present study, we found that the proportion of Gli1^+^, Rankl^+^ cells increase on compression side (Appendix Figure A2C,D), indicating Gli1^+^ cells express Rankl to induce osteoclastogenesis. Additionally, Gli1^+^ cells are associated with Trap^+^ cells on compression side (Figure 1G), not differentiated into osteoclast. Although the percentage of Gli1^+^ cells do not increase on compression side, they can secrete Rankl and associate with Trap^+^ osteoclasts, suggesting the Gli1^+^ cells participate in bone resorption under compressive force through indirect pathway such as paracrine, while the underlying mechanism needs further investigation. These results uncover the heterogeneity of Gli1^+^ cells that display different phenotypes when they respond to different types of force. Our findings extend the understanding about Gli1^+^ cells and further uncover the influences of mechanical force on a specific stromal/stem cell subpopulation.

Given the PDL is the biological foundation of tooth movement, which distributes and resorbs force, the cells in PDL play a vital role in responding to force and modulating tissue remodelling.^35^ Recently, several biomarkers such as the Axin2^+^, Scleraxis^+^ and Osterix^+^ cells have been evaluated during periodontal development. For example, a study has reported that Axin2^+^ ‐mesenchymal PDL cells play a critical role in cementum growth.^36^ In addition, some in vivo studies about Scleraxis^+^ cells, which mainly refer to ligament cell lineages, have implicated that the expression of Scleraxis^+^ cells increases on the tension side during OTM.^12^, ^37^ However, which type of cell in vivo that mainly responds to orthodontic forces remains elusive. Gli1 is not only a transcription factor in sonic hedgehog (Shh) signalling, but also a mesenchymal progenitor marker, which can be detected in various organs including kidney, liver, lung, skin, bone and tooth.^11^, ^20^, ^38^ Additionally, they have been discovered progressively produced myofibroblast, osteoblasts and odontoblasts in different organs.^11^, ^15^, ^20^ Interestingly, regardless of the diverse fate of Gli1^+^ cells in different organs, they proliferate after injury and are responsible for repair process, implicating the rapid response capacity of them after stimuli, while it is still unclear whether Gli1^+^ cells respond to mechanical stimuli. Since Shh pathway plays a crucial role in bone formation,^39^ we speculate Gli1^+^ cells participate in force‐triggered bone remodelling. In the present study, we demonstrate that suppression of Gli1 inhibits force‐induced bone remodelling (Figures 2, 3) and disrupts the mechanotransduction of Yap (Figure 4C), implying Gli1^+^ cells possibly be indispensable force‐responsive cells during OTM. The results above implicate that inhibiting specific PDLC subpopulation can largely block the mechanical response of tissue. Though Gli1^+^ cells are major MSC population in long bone and craniofacial bone, they barely express classical MSC markers in vivo. Instead, they have been identified as typical MSCs in vitro by expressing classical MSC markers including CD90, CD73, CD44 and Sca1, as well as possessing the self‐renew and multiple differentiation potential.^15^, ^38^ Given the Gli1 protein is a transcription factor at a low expression level, transgenic mouse is an ideal model to amplify Gli1 signal and label Gli1+ cell. However, because the PDL in mouse is too little to obtain, it is difficult to isolate and culture mouse PDLSCs to identify whether Gli1^+^ cells are MSCs in PDL. Therefore, further efforts are needed to solve the technical limitations.

Studies have exhibited several factors that serve as mechanical sensors in cells including TGF‐β, Integrins, Runx2 and Yap (yes‐associated protein)/ Taz (transcriptional coactivator with PDZ‐binding motif).^30^, ^40^, ^41^ Among them, Yap is a classical transcription factor that transduces mechanical signals by transferring into nucleus, then activating gene expression to mediate cell behaviour.^31^ It has been proposed that tensile force associated with cytoskeletal organization leads to Yap nuclear localization and activation, promoting cell proliferation and osteogenic differentiation.^21^ Meanwhile, compressive force can result in decreased expression of Yap and facilitate osteoclast formation.^10^ However, the mechanism of how specific PDLC subpopulation respond to orthodontic force in vivo remains unclear. In this study, we showed for the first time the strong correlation between active‐Yap and Gli1 in both Gli1‐labelled and loss‐of‐function models. Importantly, specific knock of the *Yap* gene in Gli1^+^ cells led to arrest of OTM distance (Figure 5B) and bone remodelling (Figure 5F‐G), implying that Yap is the mechanical sensor in Gli1^+^ cells mediating cell behaviour and supporting OTM process. Recently, YAP has been reported as a key regulator in mediating the balance of adipogenic and osteogenic differentiation of MSCs in vitro. High level of Yap expression facilitates cell proliferation and osteogenic differentiation of MSCs, while low level of YAP activity inhibits osteogenesis differentiation of MSCs.^42^ In vivo, Yap ablation in adult mice leads to down‐regulation of osteoblast activity and defective bone formation.^43^ In the present study, the active‐Yap^+^ cells decreased under compression, illuminating that the compressive force inhibits the expression of active‐Yap, which may suppress osteogenic differentiation of PDLCs and promote bone resorption. However, the role of YAP as mechanical sensor during bone remodelling remains controversial; therefore, the specific mechanism needs further investigation.

In conclusion, these results highlighted Gli1^+^ cells play a critical role in the process of OTM, thus providing functional evidence about the mechanism of force‐mediated bone remodelling in vivo. Particularly, we first discovered Gli1^+^ cells are force‐responsive cells, identifying a novel role for Gli1^+^ cells.

## CONFLICT OF INTEREST

The authors have no conflicts of interest to declare.

## AUTHOR CONTRIBUTION

An‐Qi Liu, Li‐Shu Zhang and Ji Chen contributed equally to the study design, manuscript preparation and data collection. Bing‐Dong Sui and Jin Liu made contributions to design and data acquisition, and drafted the manuscript, Qi‐Ming Zhai and Yan‐Jiao Li contributed to animal experiment and data analysis, and drafted the manuscript. Meng Bai and Kai Chen provided the data acquisition and critically revised the manuscript. Yan Jin contributed to the study conception and critically revised the manuscript. Cheng‐Hu Hu and Fang Jin supervised the research, oversaw the collection of results and data interpretation, and critically revised the manuscript. All authors approved the final manuscript as submitted and agreed to be accountable for all aspects of the work.

## Data Availability

The data sets used and/or analysed during the current study are available from the corresponding author on reasonable request.

## APPENDIX 1

### Animals

For tracing, the distribution of Gli1^+^ cells in OTM, *Gli1‐LacZ* mice that inserted *LacZ* into the *Gli1* locus was used. For the ablation experiments, we crossed *Gli1‐CreER^T2^* mice with *ROSA26‐eGFP‐DTA* mice to generate *Gli1‐CreER^T2^*; *Rosa‐DTA* mice, which genetically ablate Gli1^+^ cells by inducing expression of the cytotoxic diphtheria toxin A (DTA) after tamoxifen (Sigma‐Aldrich, USA) treatment. To further assess the role of Yap in Gli1^+^ cells, we generated *Gli1‐CreER^T2^*; *Yap^flox^* transgenic mice to specifically knock of the *Yap* gene in Gli1^+^ cells after tamoxifen application.

### The genotyping and quantitative real‐time PCR (qRT‐PCR)

Primers used for genotyping include the following:

#### Gli1‐LacZ

oIMR8770—5ʹ‐TCT GCC AGT TTG AGG GGA CGA C‐3ʹ—Mutant Reverse

oIMR7888—5ʹ‐GGG ATC TGT GCC TGA AAC TG‐3ʹ—Common;

oIMR9034—5ʹ‐AGG TGA GAC GAC TGC CAA GT‐3ʹ—Wild‐type Reverse.

#### ROSA26‐eGFP‐DTA

oIMR8052—5ʹ‐GCG AAG AGT TTG TCC TCA ACC‐3ʹ—Mutant Reverse;

oIMR8545—5ʹ‐AAA GTC GCT CTG AGT TGT TAT‐3ʹ—Common;

oIMR8546—5ʹ‐GGA GCG GGA GAA ATG GAT ATG‐3ʹ—Wild‐type Reverse.

#### Gli1‐CreER^T2^

oIMR1084—5ʹ‐GCG GTC TGG CAG TAA AAA CTA TC −3ʹ—Transgene Forward;

oIMR1085—5ʹ‐GTG AAA CAG CAT TGC TGT CAC TT‐3ʹ—Transgene Reverse;

oIMR7888—5ʹ‐GGG ATC TGT GCC TGA AAC TG‐3ʹ—Wild‐type Forward;

oIMR7889—5ʹ‐CTT GTG GTG GAG TCA TTG GA‐3ʹ—Wild‐type Reverse.

#### Yap^flox^

29878—5ʹ‐AGG ACA GCC AGG ACT ACA CAG‐3ʹ—Forward;

29879—5ʹ‐CAC CAG CCT TTA AAT TGA GAA C‐3ʹ—Reverse.

We collected the periodontal tissue from DTA and wild‐type mice to compare the expression level of *Gli1* gene. The primers used for *Gli1* are as follows:5ʹ‐CCA AGC CAA CTT TAT GTC AGG G‐3ʹ—Forward; 5ʹ‐AGC CCG CTT CTT TGT TAA TTT GA‐3ʹ—Reverse.
